# Leveraging 13 million responses to the Facebook COVID-19 Trends and Impact Survey to examine vaccine hesitancy, vaccination, and mask wearing, January 2021-February 2022

**DOI:** 10.21203/rs.3.rs-1712246/v1

**Published:** 2022-06-10

**Authors:** Quynh C. Nguyen, Isha Yardi, Francia Ximena Marin Gutierrez, Heran Mane, Xiaohe Yue

**Affiliations:** 1 Department of Epidemiology and Biostatistics, University of Maryland School of Public Health, College Park, Maryland, United States; 2 Department of Behavioral and Community Health, University of Maryland School of Public Health, College Park, Maryland, USA

**Keywords:** COVID-19 vaccine hesitancy, COVID-19 vaccine, health surveys, big data, social media

## Abstract

**Background:**

The urgency of the COVID-19 global pandemic called upon the joint efforts from the scientific and private sectors to work together to track vaccine acceptance, prevention behaviors, and symptoms.

**Methods:**

Our study utilized individual responses to the Facebook’s COVID-19 Trends and Impact Survey from January 2021 to February 2022 (n=13,426,245) to examine contextual and individual-level predictors of COVID-19 vaccine hesitancy, vaccination, and mask wearing. Adjusted logistic regression models were developed to examine individual and zip code predictors of COVID-19 vaccine hesitancy and vaccination status. Given the COVID vaccine was rolled out in phases in the U.S. we conducted analyses stratified by time, January 2021-May 2021 (Time 1) and June 2021-February 2022 (Time 2).

**Results:**

On January 2021 only 9% of Facebook respondents reported receiving the COVID-19 vaccine, and 45% were vaccine hesitant. By February 2022, 80% of respondents were vaccinated and only 18% were vaccine hesitant. Individuals who were older, held higher educational degrees, worked in white collar jobs, wore a mask most of the time or some of the time, and identified as white and Asian had higher COVID-19 vaccination rates and lower vaccine hesitancy across Time 1 and Time 2. COVID vaccinations were lower among essential workers and blue-collar occupations (OR=0.31–0.40) including those in food preparation and serving, construction, installation and repair, transportation, and production in Time 1. In Time 2, these disparities attenuated but were still present (OR-0.36–0.64). For these same occupation groups, vaccine hesitancy was higher (OR=1.88–2.30 in Time 1) and (OR=2.05–2.80 in Time 2). By Time 2, all adults were eligible for the COVID-19 vaccine, but blacks (OR=0.71; 95% CI: 0.70–0.72) and multiracial (OR=0.47; 95% CI: 0.47–0.48) individuals had lower vaccination and higher vaccine hesitancy compared to whites.

**Conclusions:**

Associations found in earlier phases of the pandemic were generally found to also be present later in the pandemic, indicating stability in inequities. Additionally, inequities in these important outcomes suggests more work is needed to bridge gaps to ensure that the burden of COVID-19 risk does not disproportionately fall upon subgroups of the population.

## Background

On January 20th, 2020, the Centers for Disease Prevention and Control (CDC) confirmed the first case of the COVID-19 virus in the United States [1]. As of April 21, 2022, COVID-19 has globally taken over 6.2 million lives and infected over 504 million individuals [2]. The urgency of the COVID-19 global pandemic called upon the scientific community to implement public health policy measures and expedite the development and distribution of a universal vaccine.

The CDC released its first mask recommendation on April 3, 2020 to curb the spread of COVID-19 [3]. Across the duration of the pandemic, 39 states and Puerto Rico and Washington DC required people to wear masks in public. However, 11 states did not have any mask mandates at any point and some states such as Florida, Iowa, Montana, Tennessee and Texas utilized legislation or executive action to prevent local governments from implementing mask mandates. Approximately 32 states lifted indoor mask mandates after the pandemic eased in the summer of 2021 [4]. As of May 2022, no states are broadly requiring mask wearing in public, although some states mandate mask wearing in high risk settings such as healthcare and long-term care facilities [4]. Remaining mask mandates and policies vary by city and demographic requirements, such as some areas extending their mask mandates specifically for individuals below a certain age [4]. In places with no state or local mask requirements, businesses and private establishments may institute their own mask policies.

Additionally, COVID vaccines were and remain an important preventative measure. On December 11th, 2020, the Food and Drug Administration (FDA) approved the use of Pfizer-BioNTech COVID-19 vaccine under emergency use authorization for individuals 16 years and older [5]. Soon thereafter, the Moderna and Johnson & Johnson COVID-19 vaccines were approved [6]. COVID-19 vaccines help prevent infections, symptomatic illness, hospitalization, and death [7]. They also work to protect against COVID-19 variants and while vaccinated individuals may experience breakthrough infections, COVID-19 vaccines help prevent severe illness and mortality [8]. According to the CDC, as of April 2022, a total of 567 million vaccine doses were administered in the US. About 77% of the U.S population received at least one dose and 66% were fully vaccinated, and 99 million booster doses had been administered which constituted only 50% of the total booster-eligible population [9].

Vaccine hesitancy has been a palpable roadblock to getting individuals vaccinated in the United States. Vaccine hesitancy and mistrust at large has existed as a public health issue for generations [10]. Current vaccine hesitancy is often linked to landmark study published in *The Lancet* which falsely linked the incidence of autism to the measles, mumps, and rubella (MMR) vaccine [11]. This study was retracted 12-years after publication, but likely seeded and fed a large proportion of the modern COVID-19 vaccine specific hesitancy that is observed today [11]. The US Household Pulse Survey that found almost 50% of vaccine hesitant individuals were concerned about potential vaccine side effects and 40% of vaccine hesitant individuals simply did not trust the COVID-19 vaccine or harbored skepticism towards the vaccine’s efficacy [12]. The more than two year-long global pandemic has likely exacerbated traditional reasons for hesitancy observed with other vaccines [13]. The COVID-19 pandemic has largely been characterized by hostile political undertones and the spreading of misinformation which can lead to more hesitancy towards getting the vaccine. Public surveys identified political affiliation [14, 15] and government trust [16] as influencing vaccine hesitancy, with individuals citing those who endorse the vaccine [17], geographical origin of the vaccine [18], and political motives playing a role in their attitudes towards the COVID-19 vaccine.

Regarding COVID-19 vaccination mandates, only 22 states have instituted a vaccine mandate, with a majority of these states being concentrated on the east and west coasts of the country [19]. States included in this number have vaccination mandates listed either for all individuals or for certain demographics such as school employees and healthcare workers. 15 states do not have any vaccine mandate in place, while 14 states prohibit the passage of any COVID-19 vaccine mandates [19, 20].

### Study Aims and Study Hypotheses

The goal of this project is to understand COVID-19 related behaviors including COVID-19 vaccination, vaccine hesitancy, and mask wearing in the United States and explore individual-level and contextual characteristics that significantly predict these beliefs and behaviors.

This study utilized over 13 million individual responses to the Facebook’s COVID-19 Trends and Impact Survey collected from January 2021 to February 2022. We hypothesized that older individuals, females, higher education groups, and white collar occupations will have lower COVID-19 vaccine hesitancy and higher COVID-19 vaccination rates and mask wearing. At the zip code level, we hypothesized that communities with higher socioeconomic status and greater urban development will have lower vaccine hesitancy and higher COVID-19 vaccination rates and mask wearing. Additionally, given that the COVID vaccine was rolled out in phases in the United States with only certain population groups gaining access to the vaccine in stages, we examined we examine patterns in COVID-19 vaccination before and after it was available to the general population. We hope that these results can be used to inform the development of policies and programs to help protect all individuals from coronavirus.

## Methods

Facebook’s COVID-19 Trends and Impact Survey, in collaboration with Carnegie Mellon University and the University of Maryland, was developed to collect information on COVID-19 symptoms, COVID-19 testing, vaccination status, vaccine hesitancy, health behaviors, demographic and family characteristics. The survey was implemented in the United States and globally with Facebook users from over 130 countries invited to take the survey daily. In our study, we utilized survey data from the United States. We obtained the data through a restricted data access agreement with Carnegie Mellon that enabled us to have individual-level response data with zip code identifiers. We used individual survey responses from January 2021-February 2022 (n=13,426,245). Our study was approved by the Institutional Review Board at the University of Maryland College Park. Below, we provided details on survey questions utilized.

### COVID-19 vaccination and vaccine hesitancy

Respondents were asked, “Have you had a COVID-19 vaccination?” Those who responded with “yes” were coded as having received a COVID vaccine. The survey also asked respondents the number of doses they have received, but given the survey was implemented daily on different cross-sections of the United States population and our study period was from January 2021 to February 2022, not all individuals would have the opportunity to receive two or more doses. Thus, our analyses examined whether individuals received at least one dose of a COVID-19 vaccine. If participants responded “No,” they were subsequently asked “If a vaccine to prevent COVID-19 were offered to you today, would you choose to get vaccinated?” Participants were given four response options: 1. “Yes, definitely,” 2. “Yes, probably,” 3. “No, probably not,” 4. “No, definitely not.” In our analyses, participants selecting options 2–4 were coded as “vaccine hesitant” while those who responded with option 1 were categorized as “not vaccine hesitant.”

If the participant answered with anything other than “Yes, definitely” they would choose to get vaccinated, a question appeared asking them to select from the following vaccine hesitancy reasons: I am concerned about possible side effects of a COVID-19 vaccine; I don’t know if a COVID-19 vaccine will work; I don’t believe I need a COVID-19 vaccine; I don’t like vaccines; I plan to wait and see if it is safe and may get it later; I think other people need it more than I do right now; I am concerned about the cost of a COVID-19 vaccine; I don’t trust the government; It is against my religious beliefs; Other. Among those who are vaccine hesitant, we examined the top reasons reported for why respondents did not receive the COVID-19 vaccine.

### Individual Level Covariates.

Other individual-level characteristics accounted for in analyses included whether symptomatic (“In the past 24 hours, have you or anyone in your household had any of the following symptoms, fever, sore throat, cough, shortness of breath, difficulty breathing, Age (categories into the following groups 18–24 years, 25–34 years, 35–44 years, 45–54 years, 55–64 years, 65–74 years, 75 years or older), Race/ethnicity (White, Hispanic, Black, Asian, American Indian/Alaska Native, Native Hawaiian/Pacific Islander, Multiple race, Unknown race), Travel outside state (“In the past 7 days, have you traveled outside of your state?”), Occupation type (Community and social service; Education, library services; Arts, entertainment, media; Healthcare practitioners; Healthcare support; Protective service; Food preparation and serving; Building/grounds cleaning & maintenance; Personal care & service; Sales; Office & admin support; Construction; Installation & repair; Production; Transportation & material moving; Other occupation; Unemployed in past 4 weeks), User Language (English, Other), Highest Education Degree (Less than high school, High school graduate or equivalent(GED), Some college, 2 year degree, 4 year degree, master’s degree, Professional degree, Doctorate), Gender (male, female, others), Family size (number of people in household), Mask use (“In the past 7 days, how often did you wear a mask when in public? “1”:”All the time”,”2”:”Most of the time”,”3”:”Some of the time”,”4”:”A little of the time”,”5”:”None of the time”,”6”:”I have not been in public during the past 7 days”).

### Zip Code Level Variables

Analyses also examined the association between neighborhood characteristics (operationalized at the zip code level) and COVID-19 health behaviors. Zip code level variables were obtained from the American Community Survey (ACS) 2018 5-year estimates and included median age, median household income, percentage black, percent Hispanic, percentage with a bachelor’s degree, and civilian employment rate. Zip code built environment characteristics were created utilizing computer vision on Google Street View images. Images were processed using trained Visual Geometry Group (VGG-19 model) deep convolutional networks (previously detailed by Nguyen et al. [21–23]) to identify the built environment features of interest which included presence of sidewalk and mixed land use (mixture of buildings other than detached single family homes) with accuracies of 85% for sidewalks and 82% for mixed land use.

#### Analytic Approach

We estimated the prevalence of vaccine hesitancy among survey respondents and top vaccine hesitancy reasons. We graphed temporal trends in vaccine hesitancy, vaccination status, and mask wearing. Adjusted logistic regression models were developed to examine predictors of COVID-19 vaccine hesitancy and vaccination status, controlling for individual level and zip code level potential confounders. Regression analyses were run separately for two time periods; January 2021-May 2021 (Time 1) and June 2021-February 2022 (time 2). Time 1 was characterized by greater limitations in COVID eligibility and vaccine supplies. Time 2 saw individuals 5 years and older qualify for the COVID vaccine and more availability in COVID vaccination. All survey analyses were weighted to correct for sampling bias.

## Results

Table 1 presents descriptive statistics of survey respondents from January 2020 to February 2022. Respondents came from a variety of age groups with seemingly adequate representation from the younger and older groups. For example, 26% of respondents were 18–35 years old and 22% were 65 years and older (Table 1). About 40% had a bachelor’s degree or higher. 52% were female, 44% were male and 4% reported other gender. About 7% reported a user language other than English. About 4% worked in the food industry, 5% in education, and 7% in healthcare. About 43% reported not having worked for pay in the past 4 weeks.

**Figure 1.**
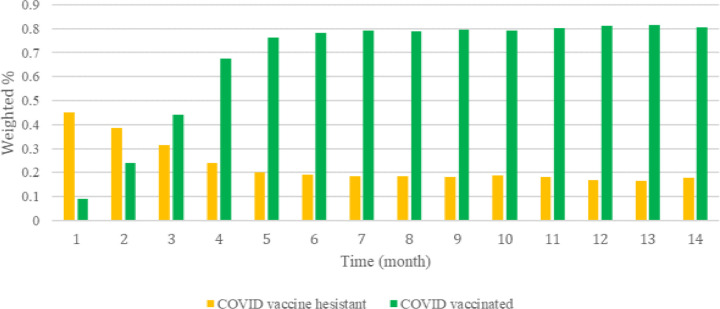
COVID vaccination and COVID vaccine hesitancy by time, January 2021-Febrary 2022. COVID vaccinated were Facebook survey respondents who reported having gotten at least one dose of a COVID-19 vaccine. COVID vaccine hesitant were individuals who indicated that they would “No, probably not” and “No, definitely not” get the COVID vaccine if it was offered to them today. X-axis indicates study month; Month 1 is January 2021 and Month 14 is February 2022.

Figure 1 displays temporal trends in COVID vaccinations and COVID vaccine hesitancy. On January 2021 (month 1 of our study), about 9% of respondents had received at least one dose of a COVID vaccine. COVID vaccinations increased quickly to 44% by March 2021 and 68% by April 2021. By February 2022 (month 14), COVID vaccinations had reached 80% among Facebook respondents. Across the study time period, we saw COVID vaccine hesitancy decrease. On January 2021, 45% reported being COVID vaccine hesitant. By March 2021, vaccine hesitancy had decreased to 31% and was 24% in April 2021. It continued to decrease and by Feb 2022 it was 18% (Figure 1).

**Figure 2.**
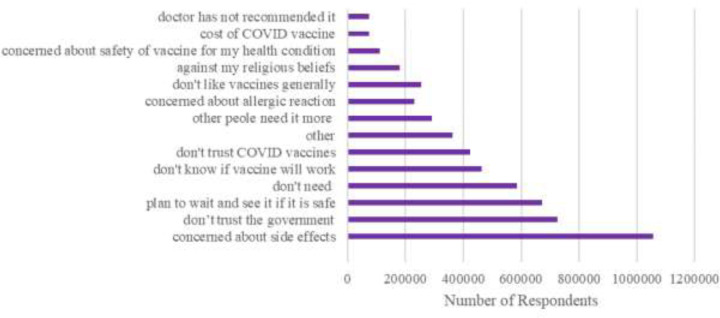
COVID-19 Vaccine Hesitancy Reason, Jan 2021-Feb 2022. Hesitancy reasons among vaccine hesitant individuals. Respondents could select multiple reasons.

Among the vaccine hesitant, the top five reasons for being hesitant about getting the COVID-19 vaccine include the following: concerned about vaccine side effects (19%), not trusting the government (13%), planned to wait and see whether the vaccine is safe (12%), believed they did not need the vaccine (11%), and don’t know if the vaccine will work (8%) (Figure 2).

**Figure 3.**
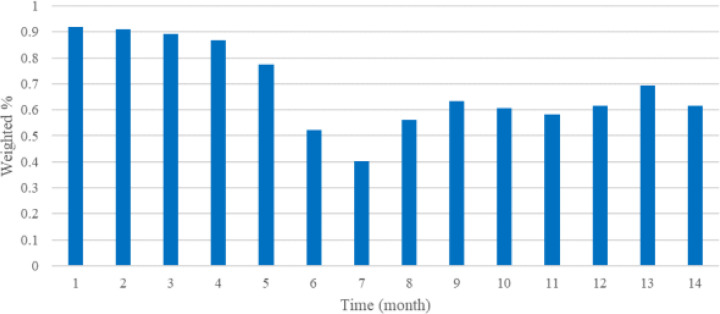
Mask wearing most/all the time in public (Jan 2021-Feb 2022). Temporal trends in the prevalence of Facebook survey respondents reporting they were masks most or all of the time in public. X-axis indicates study month; Month 1 is January 2021 and Month 14 is February 2022.

Masking-wearing fluctuated across the time period. In January 2021, 91% reported wearing masks most or all the time in public. Mask wearing steadily decreased as COVID vaccination increased and was 40% in July 2021. As COVID variants emerged, mask wearing increased and was 62% in February 2022 (Figure 3).

Table 2 displays adjusted logistic regression model results. Data suggests that older people were more likely to be vaccinated: for example, respondents who were 65 years or older were 3–5 times more likely to be fully vaccinated compared to those aged 18–24 years old. The data also suggests that older people were less likely to show COVID-19 vaccine hesitancy: respondents who were 75 years or older had an odds ratio as low as 0.21–0.24 (Time 1, Time 2 respectively) which means that they were 76–79% less likely to show vaccine hesitancy compared to those aged 18–24 years old. Respondents who had a bachelors’ degree or higher were more likely to be vaccinated than lower education groups and they were about 60% less likely to COVID-19 vaccine hesitant. Respondents who worked in the healthcare industry were more likely to be vaccinated and showed less vaccine hesitancy in Time 1. However, this relationship disappeared in Time 2. At Time 1, COVID vaccinations were lower among essential workers and blue-collar occupations (OR=0.31–0.40) including those in food preparation and serving, construction, installation and repair, transportation, and production (Table 2). In Time 2, these disparities attenuated but were still present (OR-0.36–0.64). For these same occupation groups, vaccine hesitancy was higher (OR=1.88–2.30 in Time 1) and (OR=2.05–2.80 in Time 2) (Table 2)

Across the two time periods, wearing masks a little of the time (OR=0.51–0.55) or none of the time (OR=0.19–0.28) were associated with lower COVID vaccination. Wearing masks a little of the time or none of the time was associated with 6–12 times higher odds of vaccine hesitancy in Time 1 and 2–6 times higher odds in Time 2 (Table 2). The majority of zip code level contextual characteristics did not strongly predict COVID vaccination status or vaccine hesitancy. The strongest predictor was zip code level percentage of the population with a bachelors’ which was associated with higher vaccination and lower vaccine hesitancy.

## Discussion

Study findings indicate that age was a major predictor for COVID-19 vaccine uptake, with higher odds of vaccine uptake increasing with age. Older age groups are likely to have more comorbidities, underlying health conditions, and physiological changes that accompany the aging process. The increased risk of severe illness from COVID-19 may incentivize older adults to take the COVID-19 vaccine as a preventative health measure, reflected higher COVID-19 vaccine uptake and lower vaccine hesitancy [24]. Additionally, older adults were among the first groups eligible to receive the COVID-19 and thus early access may have assisted with vaccine uptake. According to a report by the CDC, individuals between the ages of 5 and 11 had the lowest rates of vaccinations, with 33.6% have received at least one dose and 26.6% have been fully vaccinated [25]. On the other hand, individuals older than 65 years presented the highest rates of vaccination. 95.0% of individuals between the ages of 65 and 74 have received at least one dose, 91.2% have been fully vaccinated and 65.2% have received the booster dose. Similarly, 95.0% of individuals ages 75+ have received at least one dose, 85.5% have been fully vaccinated and 68.9% have received the booster dose.

Consistent with previous studies’ findings [26], we also found that educational attainment was a significant positive predictor for COVID-19 vaccine uptake. Individuals with higher educational attainment may have better access to accurate vaccine information and the health literacy to understand that health information and navigate initially complex systems for obtaining the vaccine. In a survey done by the Census in December 2021, 49.6% of individuals were concerned with the vaccine side effects, 42.4% of individuals did not trust the vaccine and 35.4% of individuals did not trust the government [27]. Individuals with higher educational attainment may also be able to better understand how the vaccine works, which can reduce fear surrounding possible side effects of the vaccine. According to a Census report, the unvaccinated adults who were most hard to reach were more likely to be young adults under the age of 50, non-white, and unmarried [25]. These adults presented lower levels of education and economic stability, tending to manifest as increased difficulty meeting daily expenses. They were also more likely to report disabilities such as difficulty seeing, hearing, transporting, remembering, or having complete impairment, which made it harder for them to access the vaccine.

In our study, occupation was also a major predictor for COVID-19 vaccine uptake. Those who worked in the healthcare industry as a practitioner or supporter were significantly more likely to have received one or more doses of the COVID-19 vaccine than those who worked in other professional roles. Healthcare workers were on the front lines during the initial stages of the pandemic, and were in the highest priority group to receive the vaccine when it was first being distributed. The daily exposure to high-risk individuals and severe COVID-19 presentations may have pushed those working in healthcare to take the vaccine readily when it was offered to them [28]. Many hospitals and healthcare facilities also instituted a vaccine mandate for all employees, which may explain the significant association between those working in healthcare and vaccine uptake. As of September 21, 2021, at least 174 health systems required all of their employees to be vaccinated against COVID-19 [29], and a recent ruling by the Supreme Court enabled the Centers for Medicare and Medicaid Services to require all Medicare and Medicaid service providers to be vaccinated [30]. This may explain how the significant association between vaccination and working in healthcare is consistent in both from January 2021 to May 2021 and June 2021 to February 2022. Those who worked in arts, entertainment, and media also demonstrated a significant increase in vaccine uptake. As the pandemic waned and media production went back to in-person work, many large entertainment companies required that their employees be vaccinated and routinely tested to ensure worker safety, including The Walt Disney Company and NBC Universal [31]. This may have contributed to the associations observed between vaccine uptake and working in arts, entertainment, and media industries.

Those who worked in primarily blue-collar industries such as construction, installation & repair, and transportation & material moving demonstrated low rates of vaccine uptake and high rates of vaccine hesitancy across both time periods observed. A study by Carnegie Mellon University in collaboration with the University of Pittsburgh found that high-hesitancy occupations such as construction reported a lack of trust in both the COVID-19 vaccine and the U.S. government as a key driver for hesitancy. These individuals also expressed the belief that they do not need the vaccine, which may be due to the fact that much work in construction, repair, and transportation occurs either primarily outdoors or in uncrowded settings [32]. These occupations oftentimes do not need a college education to pursue, which may lead to similar hesitancy trends as those seen for educational attainment.

Moreover, our study found differential rates of vaccination and vaccine hesitancy across racial/ethnic groups. Whites and Asians were more likely to be vaccinated and have lower rates of vaccine hesitancy in comparison with blacks, multiracial individuals as well as Native American/Alaska Native, and Native Hawaiian/other Pacific Islanders. These differences could be due to differences in vaccination access and distribution. Initially neighborhoods with greater shares of whites and Asians had higher vaccination rates [33, 34]. Our results are in alignment with CDC published vaccination rates that report Asians having the highest rate of fully vaccinated (59.8%) and proportion of booster doses (65.4%), and Blacks having the lowest rate of fully vaccinated (40.6%), and the Hispanic/Latino population having the lowest proportion of booster doses (38.5%) [9].

Also, we found that individuals who identify as “other gender” had lower vaccination rates and higher rates of vaccine hesitancy than females and males. Individuals who identify with genders different than female and male had historically encountered challenges when accessing, trusting, and obtaining health care services. Thus, not trusting the COVID-19 vaccine and the system could be the reason why they presented lower rates of vaccination and higher rates of vaccine hesitancy. However, other studies have not found differences in vaccination or vaccine confidence by gender identity [33]. Exploring vaccination patterns among gender minorities is highly understudied and warrants further investigation.

## Conclusions

Findings have demonstrated that there are a variety of factors that influence individuals’ COVID-19 vaccine uptake and vaccine hesitancy. Some of these factors have been attributed to a variety of individual and community characteristics. Major predictors for COVID-19 vaccine uptake include age, education, occupation, race/ethnicity, language, and community socioeconomic status. Associations found in earlier phases of the pandemic were generally found to also be present later in the pandemic, indicating stability in inequities. Inequities in these important outcomes suggests more work is needed to bridge gaps to ensure that the burden of COVID-19 risk does not disproportionately fall upon certain subgroups.

## Figures and Tables

**Table 1.** Descriptive Statistics of Facebook Symptom Survey Respondents, Jan 2021-Feb 2022

*Respondent characteristics*	N	% (95% CI)/ Mean (SD)
COVID-19 vaccine hesitant	12,944,975	22.90 (22.86, 22.93)
COVID-19 vaccinated (at least 1 dose)	13,058,980	67.33 (67.29, 67.36)
Age 18–24	11,679,657	10.26 (10.22, 10.29)
Age 25–34	11,679,657	15.78 (15.75, 15.81)
Age 35–44	11,679,657	16.58 (16.55, 16.60)
Age 45–54	11,679,657	17.18 (17.16, 17.21)
Age 55–64	11,679,657	17.95 (17.93, 17.98)
Age 65–74	11,679,657	15.17 (15.14, 15.19)
Age 75 or older	11,679,657	7.09 (7.07, 7.10)
< High school	11,506,734	4.39 (4.37, 4.40)
High school	11,506,734	18.79 (18.76, 18.82)
Some college	11,506,734	25.30 (25.26, 25.33)
2 year degree	11,506,734	11.05 (11.03, 11.07)
4 year degree	11,506,734	22.83 (22.80, 22.86)
Master’s degree	11,506,734	3.12 (3.11, 3.14)
Professional degree	11,506,734	2.46 (2.45, 2.47)
Doctorate	11,506,734	12.06 (12.04, 12.08)
Has COVID-like symptoms	13,824,925	21.08 (21.05, 21.10)
Male	11,721,409	44.38 (44.34, 44.42)
Female	11,721,409	51.29 (51.25, 51.33)
Other gender	11,721,409	4.33 (4.31, 4.35)
White	13,843,328	54.08 (54.05, 54.12)
Hispanic	13,843,328	13.81 (13.79, 13.84)
Black	13,843,328	5.40 (5.38, 5.41)
Asian	13,843,328	2.39 (2.37, 2.40)
American Indian/Alaska Native	13,843,328	0.74 (0.74, 0.75)
Native Hawaiian/Pacific Islander	13,843,328	0.20 (0.20, 0.21)
Multiple race	13,843,328	4.44 (4.43, 4.46)
Unknown race	13,843,328	18.93 (18.90, 18.96)
Other language vs. English	13,843,328	7.27 (7.25, 7.29)
Community and social service	11,193,962	2.23 (2.22, 2.24)
Education, library occupation	11,193,962	4.73 (4.71, 4.74)
Arts, entertainment, media	11,193,962	1.82 (1.81, 1.83)
Healthcare practitioners	11,193,962	4.50 (4.49, 4.52)
Healthcare support	11,193,962	2.98 (2.97, 2.99)
Protective service	11,193,962	0.88 (0.87, 0.89)
Food preparation and serving	11,193,962	3.77 (3.75, 3.79)
Building/grounds cleaning & maintenance	11,193,962	1.34 (1.33, 1.35)
Personal care & service	11,193,962	1.14 (1.13, 1.14)
Sales	11,193,962	4.99 (4.97, 5.01)
Office & admin support	11,193,962	6.06 (6.05, 6.08)
Construction	11,193,962	1.45 (1.44, 1.46)
Installation & repair	11,193,962	2.02 (2.01, 2.04)
Production	11,193,962	1.65 (1.64, 1.66)
Transportation & material moving	11,193,962	2.60 (2.58, 2.61)
Other occupation	11,193,962	15.03 (15.01, 15.06)
Unemployed in past 4 weeks	11,193,962	42.81 (42.77, 42.85)
Travelled outside the state	11,703,670	13.17 (33.81)
Wear mask all the time	12,305,558	48.98 (48.95, 49.02)
Wear mask most of the time	12,305,558	17.02 (16.99, 17.04)
Wear mask some of the time	12,305,558	9.61 (9.59, 9.63)
Wear mask a little of the time	12,305,558	7.32 (7.30, 7.34)
Wear mask none of the time	12,305,558	14.16 (14.13, 14.18)
Have not been in public	12,305,558	2.92 (2.91, 2.93)
Family size	13,426,245	3.59 (5.54)
*Zip code characteristics*		
Population density	12,808,117	3329 (8374)
Median age	12,808,117	50.83 (2.70)
Median income	12,808,117	64178 (25449)
% of population holding a BA	12,808,117	0.31 (0.16)
Employment rate for civilians	12,808,117	0.94 (0.03)
% Black	12,808,117	0.11 (0.16)
% Hispanic	12,808,117	0.17 (0.20)
% Asian	12,808,117	0.05 (0.08)
Sidewalks	12,808,117	0.39 (0.27)
Mixed land use	12,808,117	0.27 (0.20)

**Table 2.** Time stratified models of predictors of COVID-related outcomes

*Respondent characteristics*	At least one dose of COVID vaccine		Vaccine hesitancy	
	Jan to May 2021	June to February 2022	Jan to May 2021	June to February 2022
	OR (95% CI)	OR (95% CI)	OR (95% CI)	OR (95% CI)
Age 18–24	1.00	1.00	1.00	1.00
Age 25–34	1.12 (1.10, 1.14)	0.93 (0.91, 0.94)	1.04 (1.03, 1.06)	1.12 (1.10, 1.14)
Age 35–44	1.35 (1.33, 1.37)	1.16 (1.14, 1.18)	0.84 (0.83, 0.85)	0.90 (0.89, 0.92)
Age 45–54	1.48 (1.46, 1.51)	1.42 (1.39, 1.44)	0.71 (0.70, 0.72)	0.74 (0.72, 0.75)
Age 55–64	1.88 (1.86, 1.91)	2.06 (2.03, 2.10)	0.49 (0.48, 0.50)	0.50 (0.49, 0.51)
Age 65–74	3.66 (3.60, 3.71)	3.39 (3.33, 3.46)	0.28 (0.28, 0.28)	0.30 (0.29, 0.31)
Age 75 or older	5.10 (5.02, 5.19)	3.98 (3.89, 4.06)	0.21 (0.21, 0.22)	0.24 (0.24, 0.25)
< High school	1.00	1.00	1.00	1.00
High school	1.18 (1.16, 1.20)	1.23 (1.21, 1.26)	0.92 (0.91, 0.94)	0.85 (0.83, 0.87)
Some college	1.45 (1.42, 1.47)	1.61 (1.58, 1.64)	0.69 (0.68, 0.70)	0.67 (0.65, 0.68)
2 year degree	1.53 (1.50, 1.56)	1.62 (1.58, 1.65)	0.66 (0.64, 0.67)	0.67 (0.66, 0.69)
4 year degree	1.87 (1.84, 1.91)	2.53 (2.48, 2.59)	0.41 (0.40, 0.42)	0.43 (0.42, 0.44)
Master’s degree	2.05 (2.01, 2.09)	2.08 (2.02, 2.14)	0.36 (0.35, 0.36)	0.52 (0.51, 0.54)
Professional degree	1.84 (1.79, 1.88)	1.53 (1.48, 1.57)	0.38 (0.37, 0.39)	0.70 (0.68, 0.72)
Doctorate	2.06 (2.02, 2.09)	2.80 (2.74, 2.86)	0.33 (0.33, 0.34)	0.39 (0.38, 0.40)
Has COVID-like symptoms	0.59 (0.58, 0.59)	0.81 (0.81, 0.82)	1.41 (1.40, 1.42)	1.16 (1.15, 1.17)
Male	1.00	1.00	1.00	1.00
Female	1.03 (1.03, 1.04)	1.00 (1.00, 1.01)	1.27 (1.26, 1.28)	1.01 (1.01, 1.02)
Other gender	0.83 (0.81, 0.85)	0.53 (0.53, 0.54)	1.42 (1.39, 1.45)	1.88 (1.84, 1.91)
White	1.00	1.00	1.00	1.00
Hispanic	0.98 (0.97, 0.99)	0.93 (0.91, 0.94)	1.16 (1.15, 1.17)	1.03 (1.01, 1.04)
Black	0.86 (0.85, 0.87)	0.71 (0.70, 0.72)	1.82 (1.80, 1.84)	1.30 (1.28, 1.32)
Asian	1.31 (1.29, 1.34)	2.34 (2.24, 2.45)	0.83 (0.81, 0.85)	0.39 (0.37, 0.40)
American Indian/Alaska Native	1.09 (1.06, 1.12)	0.71 (0.69, 0.74)	1.21 (1.18, 1.25)	1.35 (1.31, 1.40)
Native				
Hawaiian/Pacific Islander	0.89 (0.84, 0.94)	0.69 (0.65, 0.74)	1.42 (1.34, 1.51)	1.34 (1.25, 1.45)
Multiple race	0.82 (0.81, 0.83)	0.47 (0.47, 0.48)	1.74 (1.71, 1.76)	2.13 (2.10, 2.16)
Unknown race	0.88 (0.86, 0.90)	0.54 (0.53, 0.55)	1.54 (1.50, 1.58)	1.77 (1.72, 1.81)
Other language vs. English	0.99 (0.97, 1.00)	1.48 (1.45, 1.51)	0.81 (0.80, 0.82)	0.57 (0.55, 0.58)
Community and social service	1.00	1.00	1.00	1.00
Education, library occupation	0.82 (0.81, 0.84)	1.30 (1.26, 1.34)	0.91 (0.89, 0.93)	0.78 (0.75, 0.80)
Arts, entertainment, media	0.53 (0.51, 0.54)	1.05 (1.01, 1.09)	0.97 (0.94, 1.00)	0.95 (0.91, 0.98)
Healthcare practitioners	1.89 (1.86, 1.93)	1.03 (1.00, 1.06)	0.72 (0.70, 0.74)	0.99 (0.96, 1.02)
Healthcare support	1.35 (1.32, 1.38)	1.05 (1.02, 1.09)	0.83 (0.81, 0.86)	0.95 (0.91, 0.98)
Protective service	0.74 (0.71, 0.76)	0.58 (0.55, 0.60)	1.46 (1.40, 1.52)	1.76 (1.69, 1.84)
Food preparation and serving	0.57 (0.55, 0.58)	0.75 (0.72, 0.77)	1.37 (1.33, 1.41)	1.31 (1.27, 1.35)
Building/grounds cleaning & maintenance	0.49 (0.48, 0.51)	0.56 (0.54, 0.58)	1.59 (1.54, 1.65)	1.77 (1.71, 1.85)
Personal care & service	0.53 (0.52, 0.55)	0.65 (0.62, 0.67)	1.48 (1.44, 1.53)	1.55 (1.49, 1.61)
Sales	0.40 (0.39, 0.40)	0.63 (0.61, 0.65)	1.74 (1.70, 1.78)	1.58 (1.54, 1.63)
Office & admin support	0.56 (0.55, 0.57)	1.01 (0.98, 1.04)	1.18 (1.16, 1.21)	0.99 (0.97, 1.02)
Construction	0.31 (0.30, 0.33)	0.36 (0.34, 0.37)	2.30 (2.22, 2.38)	2.80 (2.70, 2.90)
Installation & repair	0.34 (0.34, 0.35)	0.39 (0.37, 0.40)	2.29 (2.22, 2.37)	2.61 (2.52, 2.70)
Production	0.36 (0.35, 0.37)	0.64 (0.61, 0.66)	1.96 (1.90, 2.02)	1.56 (1.51, 1.62)
Transportation & material moving	0.40 (0.39, 0.41)	0.49 (0.48, 0.51)	1.88 (1.83, 1.94)	2.05 (1.98, 2.11)
Other occupation	0.48 (0.47, 0.49)	0.67 (0.65, 0.68)	1.43 (1.40, 1.47)	1.51 (1.47, 1.55)
Unemployed in past 4 weeks	0.48 (0.47, 0.49)	0.67 (0.66, 0.69)	1.51 (1.48, 1.54)	1.42 (1.39, 1.46)
Travelled outside the state	1.20 (1.19, 1.21)	0.84 (0.83, 0.85)	1.02 (1.01, 1.04)	1.22 (1.21, 1.24)
Wear mask all the time	1.00	1.00	1.00	1.00
Wear mask most of the time	1.30 (1.29, 1.31)	1.12 (1.11, 1.13)	1.55 (1.53, 1.56)	0.93 (0.92, 0.95)
Wear mask some of the time	1.04 (1.02, 1.05)	0.80 (0.79, 0.81)	2.99 (2.95, 3.03)	1.37 (1.35, 1.39)
Wear mask a little of the time	0.55 (0.54, 0.56)	0.51 (0.51, 0.52)	6.26 (6.15, 6.37)	2.21 (2.18, 2.24)
Wear mask none of the time	0.28 (0.27, 0.28)	0.19 (0.19, 0.19)	12.11 (11.88, 12.34)	5.97 (5.91, 6.04)
Have not been in public	0.39 (0.39, 0.40)	0.35 (0.35, 0.36)	1.94 (1.92, 1.97)	2.89 (2.83, 2.94)
Family size	0.99 (0.99, 0.99)	0.98 (0.98, 0.98)	1.01 (1.01, 1.01)	1.02 (1.02, 1.02)
*Zip code characteristics*				
Population density	0.99 (0.99, 1.00)	1.02 (1.01, 1.02)	1.01 (1.01, 1.01)	0.98 (0.98, 0.98)
Median age	0.99 (0.98, 1.00)	0.96 (0.95, 0.97)	1.03 (1.02, 1.04)	1.04 (1.03, 1.05)
Median income	0.97 (0.97, 0.97)	1.03 (1.02, 1.03)	1.03 (1.03, 1.04)	0.98 (0.98, 0.99)
% of population holding a bachelor’s degree	1.12 (1.11, 1.12)	1.37 (1.36, 1.38)	0.75 (0.75, 0.75)	0.72 (0.72, 0.73)
Employment rate for civilians	1.02 (1.02, 1.03)	1.01 (1.01, 1.02)	0.98 (0.97, 0.99)	0.98 (0.97, 0.99)
% Black	1.01 (1.00, 1.01)	0.99 (0.99, 0.99)	1.00 (1.00, 1.01)	1.01 (1.00, 1.01)
% Hispanic	1.04 (1.04, 1.04)	1.04 (1.03, 1.04)	0.95 (0.95, 0.96)	0.96 (0.96, 0.97)
% Asian	1.01 (1.01, 1.01)	1.01 (1.00, 1.01)	0.99 (0.99, 0.99)	0.99 (0.99, 1.00)
Sidewalks	1.01 (1.00, 1.01)	1.14 (1.13, 1.14)	0.94 (0.94, 0.94)	0.88 (0.88, 0.88)
Presence of apartments/commercial buildings	1.01 (1.00, 1.01)	0.97 (0.96, 0.97)	0.98 (0.98, 0.99)	1.03 (1.03, 1.04)
N	4,444,201	5,209,755	4,438,960	5,185,128
